# Low-temperature solution growth of ZnO nanotube arrays

**DOI:** 10.3762/bjnano.1.15

**Published:** 2010-12-09

**Authors:** Ki-Woong Chae, Qifeng Zhang, Jeong Seog Kim, Yoon-Ha Jeong, Guozhong Cao

**Affiliations:** 1 Department of Materials Science and Engineering, University of Washington, Seattle, WA 98195, USA; 2 Department of Materials Engineering, Hoseo University, Asan, South Korea; 3 BK21 Graduate School, Hoseo University, Asan, South Korea; 4 National Center for Nanomaterials Technology, Pohang University of Science and Technology, Pohang, South Korea

**Keywords:** growth from solutions, nanorods, nanotubes, supersaturation, ZnO

## Abstract

Single crystal ZnO nanotube arrays were synthesized at low temperature in an aqueous solution containing zinc nitrate and hexamethylenetetramine. It was found that the pH value of the reaction solution played an important role in mediating the growth of ZnO nanostructures. A change in the growth temperature might change the pH value of the solution and bring about the structure conversion of ZnO from nanorods to nanotubes. It was proposed that the ZnO nanorods were initially formed while the reaction solution was at a relatively high temperature (~90 °C) and therefore enriched with colloidal Zn(OH)_2_, which allowed a fast growth of ZnO nanocrystals along the [001] orientation to form nanorods. A decrease in the reaction temperature yielded a supersaturated solution, resulting in an increase in the concentration of OH^−^ ions as well as the pH value of the solution. Colloidal Zn(OH)_2_ in the supersaturated solution trended to precipitate. However, because of a slow diffusion process in view of the low temperature and low concentration of the colloidal Zn(OH)_2_, the growth of the (001) plane of ZnO nanorods was limited and only occurred at the edge of the nanorods, eventually leading to the formation of a nanotube shape. In addition, it was demonstrated that the pH might impact the surface energy difference between the polar and non-polar faces of the ZnO crystal. Such a surface energy difference became small at high pH and hereby the prioritized growth of ZnO crystal along the [001] orientation was suppressed, facilitating the formation of nanotubes. This paper demonstrates a new strategy for the fabrication of ZnO nanotubes on a large scale and presents a more comprehensive understanding of the growth of tube-shaped ZnO in aqueous solution at low temperature.

## Introduction

ZnO is a type of semiconductor with a wide band gap (3.37 eV) and a large exciton binding energy (~60 meV at room temperature). Nanostructured ZnO has been widely investigated during the past decades for different applications [1–14]. Among these nanostructures, tube-shaped ZnO crystals have attracted increasing interest due to their larger surface area than that of other crystal shapes, and therefore have potential for applications in photocatalysis, field emission, solar cells, and chemical sensors [15–21]. Compared with other synthetic techniques to obtain ZnO nanotubes, low-temperature solution growth process has been generally applied due to its simplicity and ease of fabrication [22–25]. However, the mechanisms that govern the tube-shaped morphology have received little attention [26–29].

In this paper, we developed a new strategy for the growth of tube-shaped ZnO crystals. This approach might obtain an aligned structure and was reproducible. We propose a mechanism with regard to the growth of ZnO tube structure, based on an experimental observation that the crystal morphology was associated with a change of temperature, reaction time, and pH of the reaction solution. It is pointed out that the formation of tube-shaped ZnO was due to a selective deposition of colloidal Zn(OH)_2_ at the edge of the (001) plane of ZnO nanorods that were formed in the beginning stage of the reaction.

## Results and discussion

**Figure 1:**
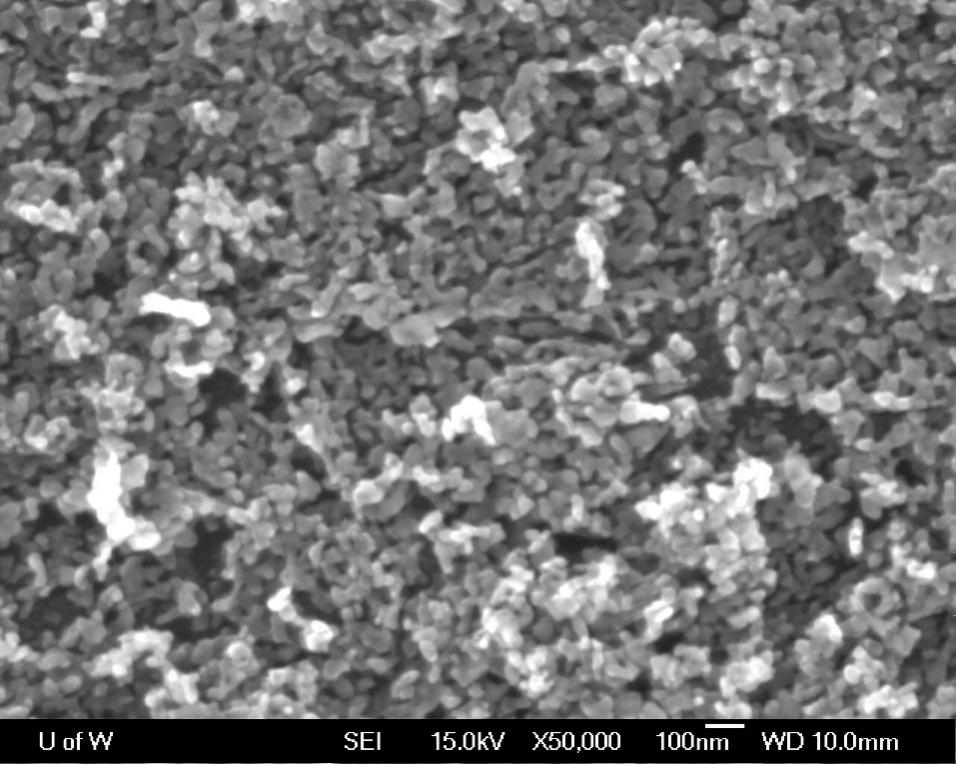
SEM image of a ZnO-seeded ITO substrate annealed at 500 °C for 30 min.

Figure 1 shows the SEM image of the film of ZnO seeds on an indium doped tin oxide (ITO) substrate prepared by electrophoretic deposition and annealed at 500 °C for 30 min. The film is homogeneous and comprises nanocrystallites of about 20–50 nm in size. It is well known that for either solution growth or vapor deposition, the morphology of ZnO nanorods in terms of size and size distribution could be significantly affected by the uniformity and crystal size of the seeds, which act as initial sites for the crystal nucleation [29–32]. The presented electrophoretic deposition method was effective for making high-quality ZnO nanocrystallite seeds on ITO substrates, as reported previously [33–35].

**Figure 2:**
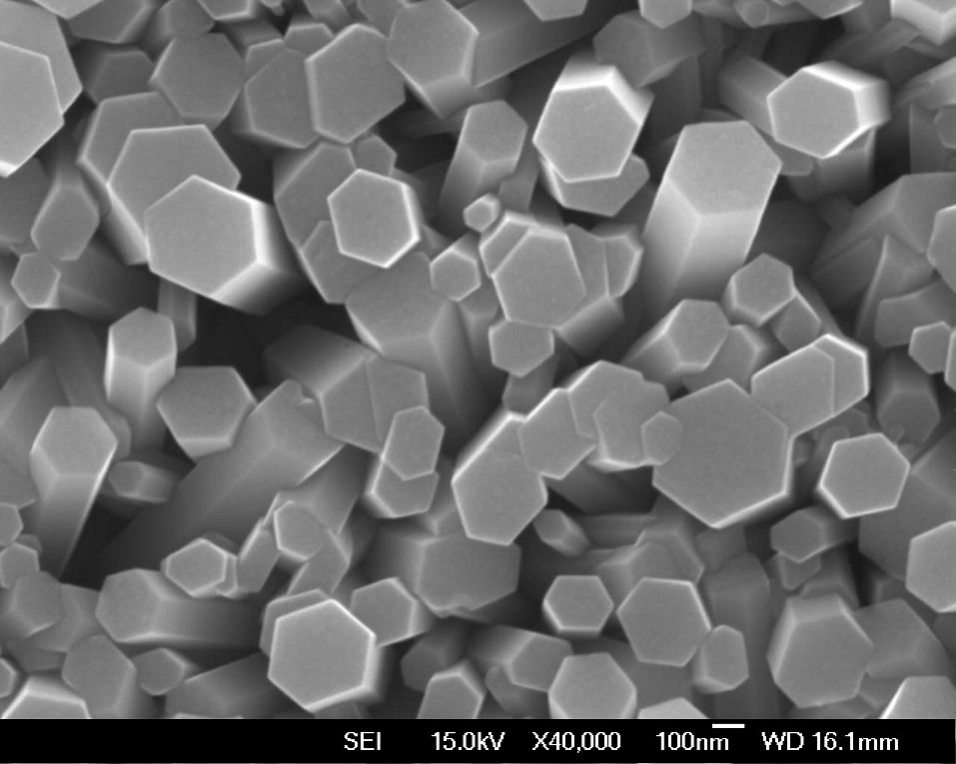
Top view of ZnO nanorod arrays grown on a ZnO-seeded ITO substrate at 90 °C for 10 h.

Figure 2 shows the SEM image of ZnO nanorod arrays obtained by a growth on the ZnO-seeded ITO substrate at 90 °C for 10 h. The synthesized ZnO nanorods with a diameter of ~200 nm were well aligned and have a perfect hexagonal shape. The length of the ZnO nanorods observed by SEM was approximately 1.2 µm. Our study also showed that if the growth time was extended to 24 h, the diameter of the nanorods might increase to ~500 nm.

**Figure 3:**
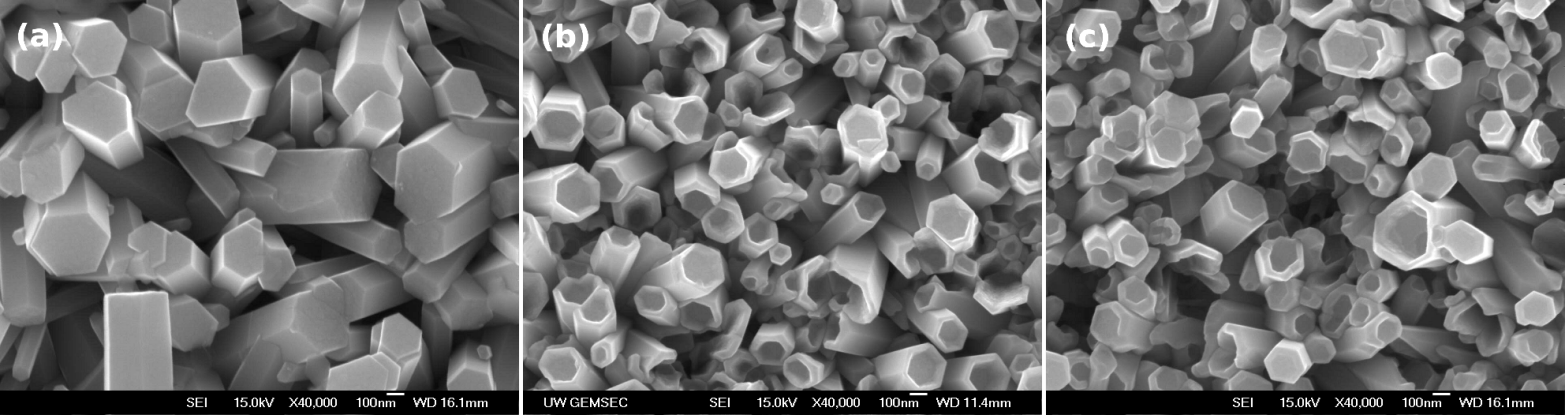
Evolution of the morphology of ZnO nanocrystals ranging from rods to tubes while the solution was kept at 90 °C for 3 h and then cooled down to (a) 80 °C (20 h), (b) 60 °C (20 h) and (c) 50 °C (20 h).

A series of interesting phenomena were observed in subsequent experiments by changing the growth time and temperature. The experiment was designed firstly to grow ZnO nanorods at 90 °C for 3 h, and then was cooled down and maintained at a certain temperature for another 20 h. The results are shown in Figure 3, corresponding to the cool-down temperatures of (a) 80 °C, (b) 60 °C, and (c) 50 °C. As shown in Figure 3a, the ZnO continually kept at 80 °C does not reveal a big difference in the structure from that of the ZnO nanorods shown in Figure 2, synthesized at 90 °C for 10 h, except for a slight difference in the size (i.e., length and diameter).

However, as shown in Figure 3b and Figure 3c, the morphologies of the samples that were temperature controlled for another 20 h growth at 60 °C and 50 °C, respectively, after a 3 hour growth at 90 °C, show that all of the ZnO nanorods had transformed drastically to tube-shaped ZnO nanocrystals. Such a behavior suggests that the decrease of the reaction temperature, after a 3 hour growth at 90 °C, is one of the important factors that cause a change in the ZnO crystal morphology. Note that the size of the ZnO nanotubes was somewhat smaller because of the lower growth temperatures (60 °C and 50 °C, shown in Figure 3b and Figure 3c, respectively) compared with those for the rod-shaped ZnO grown at higher temperatures (90 °C and 80 °C, shown in Figure 2 and Figure 3a, respectively).

Vayssieres et al. also explained the formation of ZnO microtubes by demonstrating a selective dissolution of the metastable polar (001) face of the ZnO microrods [28]. Their aging mechanism required two days to dissolute its face and convert ZnO from rods to tubes. In our experiment, the ZnO nanotubes were, however, formed by purposely decreasing the growth temperature during the synthesis, instead of relying on a long-term aging. The SEM images reveal that the top morphology of the ZnO changes from rods to tubes as the growth temperature was lowered. Sun et al. reported the formation of aligned ultrathin ZnO nanotubes on a ZnO film using a hydrothermal method [27]. They mentioned that the synthesis conditions, such as pH of the solution and Zn^2+^ concentration, might influence the relative growth rates and the stability of different crystal planes, and thus affect the morphology of ZnO nanorods. In our study, we adopted the same conditions (concentration, temperature and time) as reported [27–28]; however, the tube-shaped ZnO was not attained if we simply set a constant growth temperature during the growth process. It means that the tube-shaped ZnO structure is very sensitive to the growth conditions and it appears to be difficult to get reproducible results.

It was also reported that ZnO nanotube arrays were grown on zinc foils via a hydrothermal process, attributed to the gradient in the concentrations of zinc precursors from Zn foil to the substrate [29]. The proposed growth mechanism was based on the competition between growth rate and diffusion rate. However, our experiment cannot be explained by the gradient of ion concentration, such as Zn^2+^, since there was no concentration gradient in the solution.

**Figure 4:**
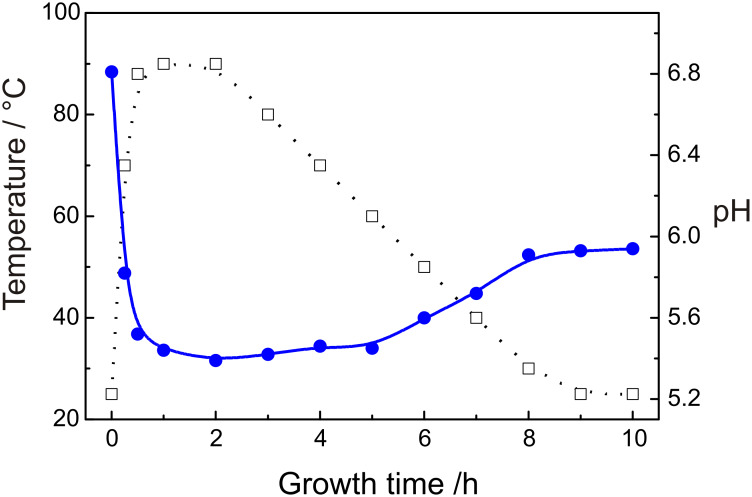
Growth temperature and measured pH value as a function of growth time for the formation of ZnO nanotubes (solid line: pH value; dotted line: growth temperature).

To understand the mechanism of the formation of tube-shaped ZnO, we investigated the dependence of the pH value of the solution on the reaction time as well as on the temperature of the solution. The results are shown in Figure 4. It can be seen that, with an increase in the solution temperature, the pH value decreased from 6.81 at 25 °C to 5.42 at 90 °C. These results are similar to the previous reports [26,36–38]. As the temperature further decreases, the pH goes up slightly and reaches 5.91 at the temperature of 25 °C. Note also that the pH values at 25 °C appear to be different before and after the reaction, i.e., the pH value (5.91) of the solution became quite lower than that (6.81) for the initial solution after cooling to 25 °C, even though it has the same initial temperature. In other words, in spite of the fall of the growth temperature, the pH value has a tendency to remain low as when the growth temperature was high (Figure 4).

**Figure 5:**
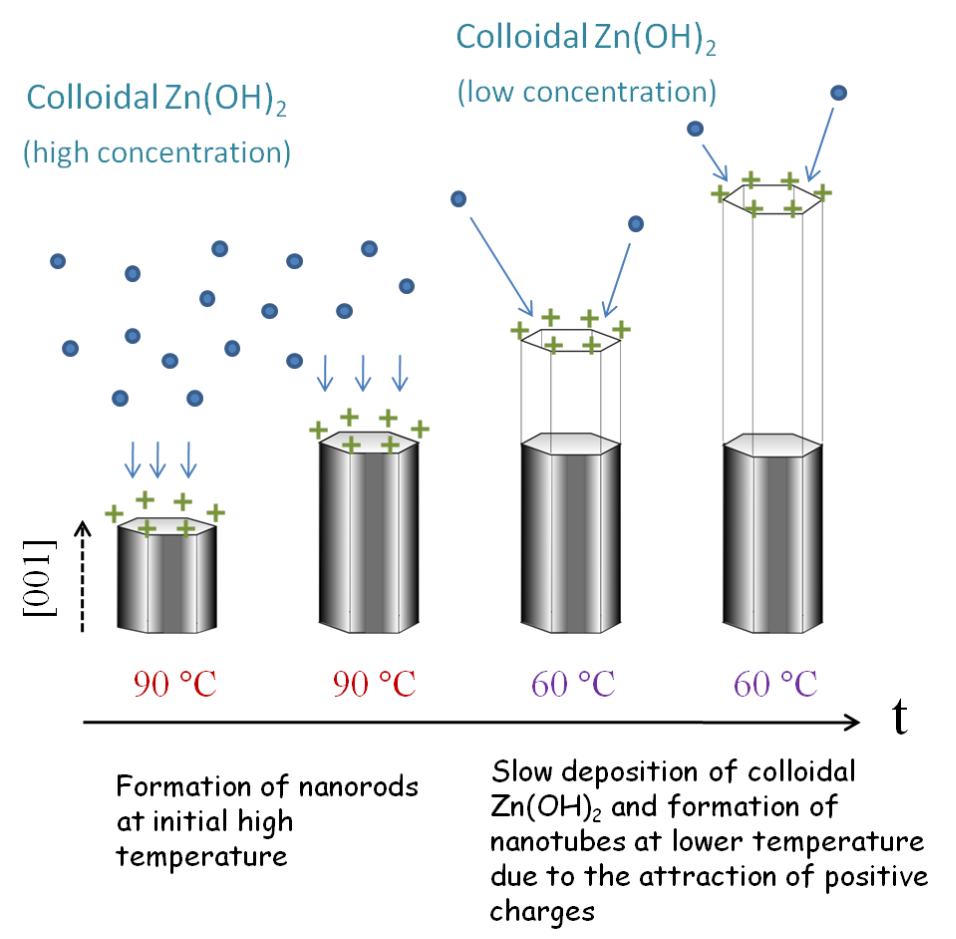
A schematic showing the evolution growth of ZnO nanocrystals from rod to tube shape as the growth temperature decreases.

Based on the fact that the pH values change as the solution temperature decreases, a mechanism of the formation of ZnO nanotubes might be proposed as follows. Firstly, ZnO nanorods with a high aspect ratio are quickly grown at 90 °C, on the condition that the reaction solution contains colloidal Zn(OH)_2_ in a high concentration. At that moment, the pH value of the solution is much lower than the isoelectric point of ZnO (~8.7–10.3) [39], implying that the ZnO crystals are positively charged [40]. This brings forth an attraction to those negatively charged species in the solution and speeds up the growth of nanorods [41]. When the growth temperature gradually cools to lower than 60 °C, the reaction solution becomes supersaturated, resulting in an increase in the concentration of OH^−^ ions as well as the pH value of the solution. In this case, colloidal Zn(OH)_2_ in the supersaturated solution tend continually to precipitate. However, because of a slow diffusion process in view of the low temperature and low concentration of the colloidal Zn(OH)_2_ (note that this is because most of the Zn(OH)_2_ has been exhausted during the high temperature stage), the growth of the nanorods is slow because of an insufficiency of precursors in the reaction solution. As the temperature becomes lower, there is a rise in the pH value of the reaction solution. However, the pH value is still below the isoelectric point of ZnO and, therefore, the ZnO nanorods are still positively charged. In contrast to the case of high temperature, in view of the relatively low concentration and slow diffusion of the colloidal Zn(OH)_2_, it is inferred that the growth of the nanorods is in turn predominated by the positive charges adsorbed on the surface of nanorods through attracting the negative species in the reaction solution. It can be assumed that the electric fields at the edge of the (001) plane of the nanorods is more intensive than that at the central part. This results in a continual growth of the nanorods at the edge and ultimately the formation of nanotubes. This process is schematically shown in Figure 5 indicating a two-step formation of the ZnO nanotubes.

The difference in surface energy of the polar and nonpolar planes of the ZnO nanorods and the dependence of the surface energy on the pH value of the reaction solution were also considered to be the reasons that gave rise to the formation of ZnO nanotubes. Theoretical calculations predicted that the surface energy of the nonpolar surface of ZnO was small, while the cleavage energy of the polar surface was predicted to be two times larger than the nonpolar surface [42]. The crystal morphology is normally determined by the minimization of the total surface energy, which is to a large extent related to the pH value of the reaction solution. In the literature it was reported that a decrease in the pH of the solution might result in the formation of individual needles or prismatic microcrystals [43]. The change of morphology of ZnO from rod-shape to needle-shape meant that the surface energy difference between polar and nonpolar faces was relatively big, and therefore, the total surface energy tended to be minimized through forming a small area of polar metastable face and a large area of the most stable nonpolar face.

**Figure 6:**
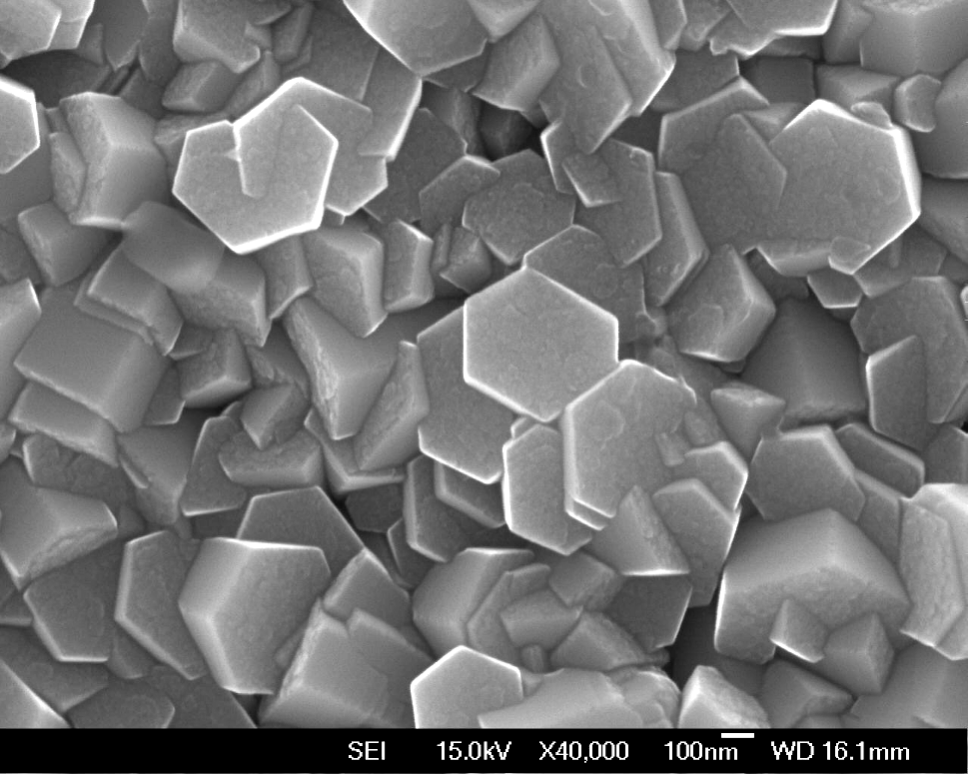
SEM top morphology of ZnO nanorod arrays grown on a ZnO-seeded ITO substrate at 60 °C for 24 h.

To verify that there is also a dependence of the surface energy on the pH value of the solution, we carried out an experiment by growing ZnO nanorods at 60 °C for 24 h. The pH value of this solution at 60 °C was 5.81, higher than the value of 5.42 at 90 °C. Figure 6 shows the result of this synthesis; typical ZnO nanorods were formed with a diameter of about 400–500 nm and there were no tube-shaped crystals. Note that the diameter of the ZnO nanorods was slightly larger than their length, ~400 nm. Compared with the ZnO morphology grown at 90 °C as shown in Figure 2, the low aspect ratio of ZnO nanorods grown at 60 °C suggest a relatively high polar surface energy, and as a result, the polar surface of ZnO was growing considerably. It is also clear that the reaction process for attachment of an atom to the interface was limited by the low temperature, so that the growth rate was slow. This is a reason that the length of ZnO nanorods in Figure 6 was smaller than that of the nanorods grown at 90 °C. All these results suggest that (1) the surface energy difference of ZnO crystal between polar and non-polar face strongly depends on the pH of the reaction solution, and (2) the growth rate greatly depends on the temperature.

A big difference in the diameter of the ZnO nanotubes shown in Figure 3b compared with the nanorods in Figure 6 can be also observed. Note that these nanotubes and nanorods were grown under almost similar growth conditions, such as the solution temperature and the growth time with the exception of the initial growing conditions, i.e., supersaturated or not, and the pH value of solution. Compared with the nanorods in Figure 6, the ZnO nanotubes in Figure 3b have smaller diameters and larger aspect ratios due to the somewhat lower pH value of the reaction solution. This, in turn, indicates a high pH value may result in a low surface energy difference between polar and non-polar faces and thus inhibit the growth of ZnO along the [001] direction.

**Figure 7:**
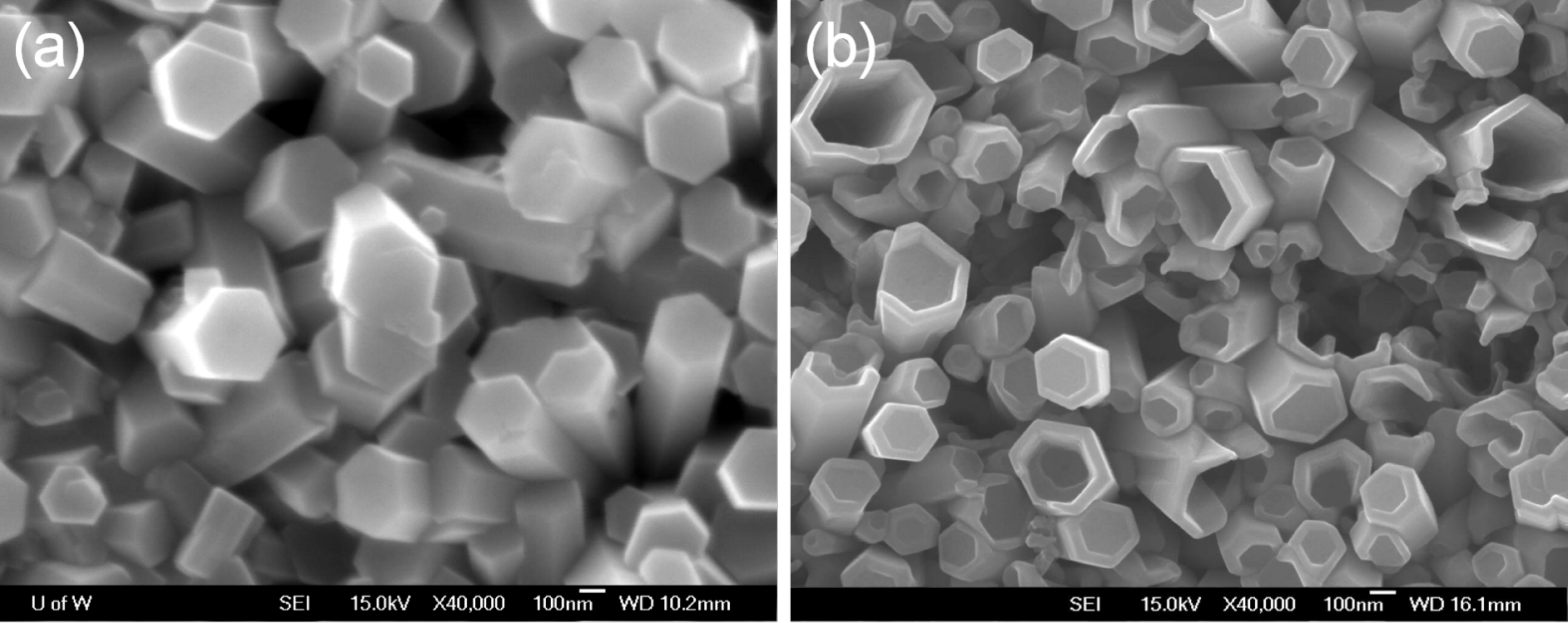
SEM images of (a) ZnO nanorods grown at 90 °C for 3 h and then 60 °C for 5 h, and (b) nanotubes grown at 60 °C for 5 h in a solution which was, however, pretreated at 60 °C for 1 h and then 90 °C for 2 h.

In a further experiment, the ZnO-seeded substrate was firstly used for growing at 90 °C for 3 h and then at 60 °C for 5 h. This experiment was designed to verify whether the ZnO nanotubes would be still obtained with a short growing time and with a low temperature growth stage. The result is shown in Figure 7a; only nanorods and no tube-shaped ZnO crystals were observed. The reason for this result can be deduced from the low pH value considering that the solution has been kept at 90 °C for 3 h. That is, the surface energy difference between polar and nonpolar surfaces are large and this facilitates the formation of nanorods which minimizes the total surface energy for the given crystal shape. Note that the ZnO nanorods in Figure 7a have diameters that are typically two times larger than that shown in Figure 3b, which means that the polar surface energy is increasing relative to low pH value. On the contrary, as shown in Figure 7b, this structure was directly grown at 60 °C for 5 h in the solution, which was pretreated to ensure a high pH value (~5.81) by holding at 60 °C for 1 h after keeping at 90 °C for 2 h, still shows tube shape. These results confirm that a high pH value at a low temperature of 60 °C could lead to continual growth of the ZnO nanorods at the edge so as to form nanotubes.

## Conclusion

ZnO nanorods can be grown in solution and changed into nanotube shape by decreasing the growth temperature during the growth process. The ZnO nanorods are formed at a relatively high temperature (~90 °C), where the reaction solution is enriched with colloidal Zn(OH)_2_ and therefore allows a fast growth of ZnO nanocrystals along the [001] orientation to form nanorods. A subsequent decrease in the temperature yields a supersaturated reaction solution, resulting in an increase in the concentration of OH^−^ ions as well as the pH value of the solution. Colloidal Zn(OH)_2_ in the supersaturated solution tends to precipitate continually. However, because of a slow diffusion process in view of the low temperature and low concentration of the colloidal Zn(OH)_2_, the growth of nanorods is limited but may still occur at the edge of the nanorods due to the attraction of accumulated positive charges to those negative species in the solution, ultimately leading to the formation of ZnO nanotubes.

The role of changing the pH value observed in the growth of ZnO crystals is shown also to have a relationship to the change of the surface energy. In the course of growing ZnO nanorods, changing the growth temperature, from a high (90 °C) to a low temperature (60 °C), leads to some change in the pH value. At the low pH value, the polar face has such a high surface energy that it permits the growth of nanorods. However, the grain growth can be inhibited by a high pH value at a low growth temperature. The competition between the change of surface energy due to pH value and growth rate dictated by the temperature can be assumed to lead to the ZnO tube structure. This investigation provides more options and flexibility in controlling methods to obtain various morphologies of ZnO crystals in terms of the change of growth temperature and pH value.

## Experimental

ZnO nanorods were grown on an indium doped tin oxide (ITO) glass substrate, on which ZnO nanocrystallites as seeds were pre-prepared via an electrophoretic deposition. Typically, the ITO substrate was immersed in a 0.5 M zinc nitrate (Fisher Scientific Corp., USA), and an electric potential of 2.5 V was applied to the ITO substrate as cathode and a platinum plate was used as the anode. The deposition time was about 5 min. The substrate was subsequently heat-treated at 500 °C for 30 min to improve the crystallinity of the film of ZnO nanocrystallites.

For the growth of ZnO nanorods, the ZnO-seeded ITO substrates were sealed in a vial containing an equimolar solution of 0.1 M zinc nitrate and hexamethylenetetramine (Alfa Aesar, USA). The solution was then heated to 90 °C and kept for 10 h in a programmable furnace (Thermolyne Type 48000, USA). A different process was adopted to form nanotubes, i.e., the solution was first kept at 90 °C for 3 h and then cooled down to a given temperature for 20 h. pH values of the reaction solution were measured every hour. The morphology of ZnO nanostructures was characterized with a scanning electron microscope (JEOL JSM7000F, Japan).
